# Ventricular strain patterns in multivalvular heart disease: a cross-sectional study

**DOI:** 10.1007/s10554-022-02737-2

**Published:** 2022-10-28

**Authors:** K. Prathiksha Prabhu, Krishnananda Nayak, Vidya Nayak, Sridevi Prabhu, V. Rekha, A. J. Ashwal, M. Sudhakar Rao

**Affiliations:** 1 Department of Cardiovascular Technology, Manipal College of Health Professions (MCHP), Manipal Academy of Higher Education, Manipal, India; 2 Department of Cardiology, Kasturba Medical College (KMC), Manipal Academy of Higher Education, Manipal, India

**Keywords:** Multivalvular heart disease, Tissue deformation imaging, Longitudinal strain, Rheumatic heart disease

## Abstract

Multivalvular heart disease (MVD) is an aggregate of regurgitant and/or stenotic lesions of at least two cardiac valves. Ventricular tissue deformation imaging is a powerful predictor of subclinical myocardial dysfunction in patients with MVD.The aim of this study was to examine the left and right ventricular strain patterns in MVD as well as observe any association between right–sided valvular involvement (tricuspid or pulmonary valve lesion) with that of aortic and/or mitral valve lesion. Patients with at least moderate forms of MVD were included in the present study. 72 patients with mean age of 56.69 ± 14.59 years and various presentations of MVD were finally enrolled in this study. The commonest cause for MVD was rheumatic heart disease in these patients. Conventional 2-dimensional parameters as well as tissue deformation imaging parameters were assessed in offline mode for these patients. The Mean ± SD values for various quantitative 2D echocardiographic conventional and tissue deformation imaging were assessed. It was observed that LV strain parameters including the global longitudinal strain (GLS) were preserved whereas the RV strain parameters were mildly reduced (RV GLS total is − 19.49 ± 6.08%). Also, when conventional echocardiographic parameters were assessed to see any association between aortic and/or mitral valve disease with that of right-sided valvular lesions (tricuspid or pulmonary); 2D conventional echocardiographic parameters like left atrial dimension (p = 0.034), TAPSE (tricuspid annular plane systolic excursion) (p < 0.001), RVSP (right ventricular systolic pressure) (p < 0.001) and IVC (inferior vena cava) dimensions (p < 0.001) showed a statistically significant result; whereas, when strain parameters for LV and RV were assessed, they did not show any statistical difference for the same. In this series of patients with significant MVD, our findings suggest that ventricular strain parameters may be reliable markers of myocardial dysfunction, but may alter depending on the underlying combination of MVD, and right ventricular strain should also be an important parameter while assessing different combinations of MVD.

## Introduction

Multivalvular heart disease (MVD) is defined as aggregate of regurgitant and/or stenotic lesions of at least two cardiac valves [1]. Valves affected in multivalvular heart disease are aortic, mitral, tricuspid and/or pulmonary. The most common associations of multiple valve lesions were aortic stenosis (AS) and mitral regurgitation (MR) and also aortic regurgitation (AR) plus mitral regurgitation (MR).

Global prevalence for MVD was reported to be 63.9 per 1, 00,000 person-years [2]. According to Euro Heart Survey, for patients presenting with MVD, the in- hospital mortality rates were 6.5% [3]. Rheumatic heart disease (RHD) is the major cause for primary or organic MVD. According to Euro Heart Survey, rheumatic fever was the most common pathogenesis accounting for about 51% of MVD cases, also other causes included degenerative valve diseases accounting for almost 41% [3]. In India, almost 44,000 patients are added every year, and expected mortality is 1.5%–3.3% per year due to RHD causing MVD [4].

Strain echocardiography/tissue deformation imaging, is one of the best imaging echocardiographic modality in diagnosis and prognostic evaluation of valvular lesions. Indication for use of strain imaging in MVD is to detect subclinical myocardial dysfunction before an overt reduction of LV ejection fraction and thus can be used to determine the exact intervention time before the ventricular function further deteriorates and also determines prognosis of MVD [5].

Most of the patients visiting our cardiology OPD present with either single (more common) or with concomitant multiple valvular lesions. Limited studies focused on ventricular loading response to multivalvular heart disease in literature. Tissue deformation imaging in identifying subclinical myocardial dysfunction among these population have not been studied thoroughly.

This study is one of its kind to emphasize ventricular strain mechanics in multiple valvular disease loading conditions. The purpose of this study was to assess left and right ventricular strain patterns in multivalvular heart disease and also to assess involvement of right sided valvular lesions with aortic and/or mitral valvular lesions.

## Materials and methods

### Study protocol and patient selection

A total of 72 patients with MVD involving more than or equal to two valves with a severity of at least more than moderate valvular lesion examined in echocardiography laboratory at the Department of Cardiology, KMC, Manipal enrolled from March 2020 to May 2021 were considered eligible for the study. Exclusion criteria included any associated ischemic heart disease, LV ejection fraction < 50%, previous history or intracardiac/prosthetic heart surgery and restrictive cardiomyopathy(RCM).The study protocol was intended to have sample size of 100 as we wished to take samples based on time bound pattern;i.e., 5 to 10 patients per month. The study was approved by the Board of Ethics Committee, Kasturba Hospital, Manipal (contact no.0820–292371) and also the subjects gave the informed consent.

### Clinical data

Clinical data included age, gender, height, weight, body mass index, history of hypertension and diabetes, smoking or drinking status, food habits and history of coronary artery disease of the subjects. Also, surface electrocardiographic (ECG) recordings of the subjects were taken and reported.

### Echocardiography

**Fig. 1:**
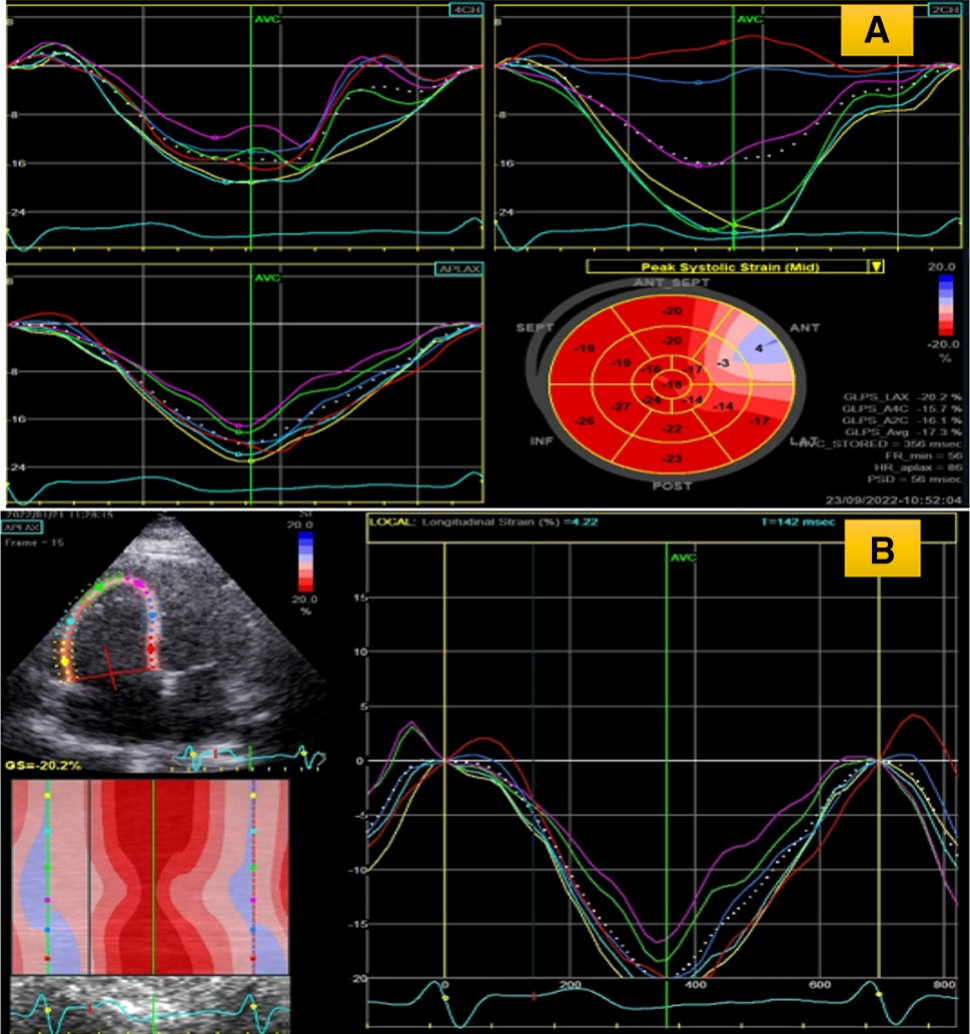
Transthoracic echocardiographic and 2D strain analysis in one of the present study’s patient with MVD (Moderate mitral stenosis and regurgitation + severe aortic regurgitation + moderate tricuspid regurgitation; (**A**)**.** Tissue deformation imaging showing LV global longitudinal strain of (− 17.3%) and (**B**)**.** Tissue deformation imaging showing RV strain of − 20.2%

At entry in the study, all patients underwent a comprehensive echocardiography using commercially available equipment (VIVID S60 echocardiographic system, GE Healthcare) at rest. Standard views were obtained with patient positioned in left lateral decubitus position. Standard views (i.e. parasternal long axis and short axis at three levels of LV and apical 4, 5, 3 and 2 chamber) views were recorded and stored LVEF measurements are taken by M mode and Simpson’s method. Left ventricular (LV) end diastolic dimensions (IVS thickness, LV posterior wall thickness and LV internal dimension) were acquired from the parasternal long axis view at the mitral valve leaflet tips. TAPSE values obtained from M mode at tricuspid valve annulus. 2 dimensional tissue deformation imaging of LV and RV strain parameters were measured offline in an echocardiography core laboratory using dedicated commercial software system (Echo-Pac) (Fig. 1). Three consecutive cardiac cycles were acquired in apical 4ch, 2ch, 3ch and short axis at 3 levels (base, mid, apex) for LV and for RV strain was measured at 3 levels of free wall (base, mid ,apex) respectively. Analysis was done by tracing the endocardial border and the region of interest is split in six segments automatically by the software. After the region of interest is selected the software spontaneously displays the strain and strain rate curve patterns of different segments along with Bull’s eye of 17 segments. Strain rates at peak systole, early diastole and late diastole were obtained.

### Data analysis

All data were entered in excel sheet. Statistical analysis was carried out using the EZR (32 bit) version 1.37. Continuous variables was expressed as Mean ± SD and compared using one way analysis of variance. Categorical data was expressed as frequency, percentage and analyzed using Chi Square test. Results were considered significant with p value less than 0.05.

## Results

### Patient characteristics

All patients who met the inclusion criteria and met no exclusion criteria were included for analysis on the basis of their diagnosis. This study included 72 patients with MVD with mean age of 56.69 ± 14.59 years, among them 34(42.7%) were males and 38(52.8%) were females, and other baseline demographic and clinical characteristics are mentioned in (Table 1).

**Table 1:** Baseline demographic and clinical characteristics of the study population

Demographic characteristics (N = 72)	Mean ± SD	N
Age (years)	56.69 ± 14.59	72
Height (cm)	165.0 ± 5.32	60
Weight (kg)	57.1 ± 10.13	60
BMI (kg/m^2^)	20.9 ± 3.28	60

Clinical characteristics (N = 72)	Group	Frequency (%)
Gender	Male	34 (42.7%)
	Female	38 (52.8%)
Hypertension	Yes	21 (29.2%)
	No	51 (70.8%)
Diabetes mellitus	Yes	12 (16.7%)
	No	60 (83.3%)
Alcohol consumption	Yes	2 (2.8%)
	No	70 (97.2%)
Smoking	Yes	3 (4.2%)
	No	69 (95.8%)
Food habits	Vegetarian	23 (31.9%)
	Non-vegetarian	49 (68.1%)
Coronary artery disease (mild)	Yes	11 (15.3%)
	No	61 (84.7%)
ECG findings	Normal sinus rhythm	48 (66.7%)
	Atrial fibrillation	24 (33.3%)
Cause of MVD	RHD	62 (83.8%)
	Degenerative disease	10 (13.5%)

*BMI* Body mass index, *ECG* Electrocardiography, *MVD* Multivalvular disease, *RHD* Rheumatic heart disease

### Multivalvular disease types of presentation and 2D conventional and tissue deformation imaging parameters of the study population

**Fig. 2:**
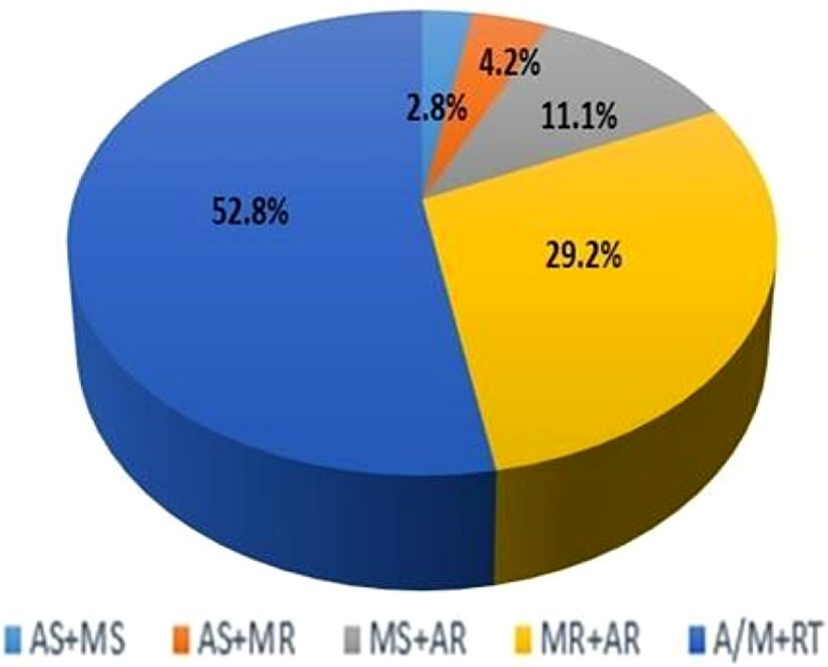
Different patterns of MVD

Out of these 72 patients included in the present study, majority of patients presented with left-sided valvular lesions (aortic and/or mitral) along with concomitant right- sided valvular lesions (tricuspid/pulmonary); remaining patients had AR and MR, AR and MS, AS and MR, AS and MS respectively (Fig. 2). The conventional 2D echocardiographic parameters were assessed for LV and RV, the results were within the normal range for both.LV and RV tissue deformation imaging parameters were also assessed like global strain, peak systolic strain, systolic strain rate, early and late diastolic strain rates respectively. The LV strain parameters were well preserved; whereas RV global strain was mildly reduced (Table 2).

**Table 2:** 2D conventional and tissue deformation imaging parameters of the study population

Parameters	Mean ± SD		N
LV EDD (cm)	4.68 ± 0.73		72
LV ESD (cm)	3.03 ± 0.58		72
LV EF (%)	63.83 ± 4.84		72
LV FS (%)	34.67 ± 3.56		72
LV EDV (ml)	74.01 ± 26.52		72
LV ESV (ml)	28.22 ± 12.22		72
LV ALEF (%)	62.92 ± 5.09		72
LA (cm)	4.01 ± 0.81		72
Aorta root (cm)	2.72 ± 0.37		72
TAPSE (cm)	1.79 ± 0.27		72
RVSP (mm Hg)	42.79 ± 21.25		72
IVC (cm)	Maximum	1.51 ± 0.43	72
	Minimum	0.82 ± 0.43	70

Parameters (LV segments) (N = 72)	Mean ± SD
GLS (%)	− 17.49 ± 41.17
Peak systolic strain (%)	− 19.64 ± 20.44
SSR (s^−1^)	− 3.14 ± 11.02
EDSR (s^−1^)	1.64 ± 1.60
LDSR(s^−1^)	0.87 ± 0.36

Parameters (RV free wall strain-basal,mid,apical) (N = 72)	Mean ± SD
LS (%)	− 19.49 ± 6.08
Peak systolic strain (%)	− 18.20 ± 6.50
SSR (s^−1^)	− 1.34 ± 0.40
EDSR (s^−1^)	1.42 ± 0.69
LDSR (s^−1^)	1.35 ± 0.70

*LV EDD* Left ventricular end diastolic dimensions, *LV ESD* Left ventricular end systolic dimensions, *LVEDV* Left ventricular end diastolic volume, *LVESV* Left ventricular end systolic volume, *LV EF* Left ventricular ejection fraction, *LV FS* Left ventricular fractional shortening, *LV ALEF* Left ventricular area-length ejection fraction, *LA* Left atrium, *TAPSE* Tricuspid annular pre-systolic excursion, *RVSP* Right ventricular systolic function, *IVC* Inferior vena cava, *LS* Longitudinal strain, *SSR* Systolic strain rate, *EDSR* Early diastolic strain rate, *LDSR* Late diastolic strain rate, *LV* Left ventricle, *RV* Right ventricle

### Association of right -sided valvular lesions (tricuspid or pulmonary disease) with 2D conventional and speckle- tracking strain echocardiographic parameters

Among 72 patients with MVD who were included in our study as per the inclusion criteria, a majority 38 patients had right -sided valvular lesions along with mitral and/or aortic valvular disease. As mentioned in (Table 3), various echocardiographic parameters were assessed to observe association with right–sided valvular lesions. It was observed that left atrial dimensions (p = 0.034) was statistically significant; TAPSE (p < 0.001), RVSP (p < 0.001) as well as IVC maximum and minimum dimensions (p < 0.001) show a highly significant statistical difference; whereas all other echocardiographic parameters are statistically non- significant.

**Table 3:** Association of right -sided valvular lesions (tricuspid or pulmonary disease) with 2D conventional and tissue deformation imaging parameters

Parameters	Right–sided valvular lesion involvement-PRESENT (Mean ± SD)		N	Right –sided valvular lesion involvement-ABSENT (Mean ± SD)	N	p value
LV EDD(cm)	4.60 ± 0.82		38	4.78 ± 0.64	33	0.303
LV ESD(cm)	3.01 ± 0.70		38	3.05 ± 0.45	33	0.763
LV EF (%)	62.95 ± 5.86		38	64.67 ± 3.08	33	0.121
LV FS (%)	34.08 ± 4.18		38	35.18 ± 2.51	33	0.176
Aorta root(cm)	2.69 ± 0.35		38	2.75 ± 0.41	33	0.491
**LA(cm)**	**4.20 ± 0.83**		**38**	**3.79 ± 0.75**	**33**	0.034*
LV EDV(ml)	74.18 ± 27.10		38	73.48 ± 26.59	33	0.913
LV ESV(ml)	29.16 ± 13.33		38	27.12 ± 11.12	33	0.48
LV ALEF (%)	62.11 ± 5.92		38	63.79 ± 3.9	33	0.158
**TAPSE(cm)**	**1.67 ± 0.33**		**38**	**1.92 ± 0.07**	**33**	< 0.001**
**RVSP(mm Hg)**	**55.56 ± 22.04**		**38**	**28.61 ± 5.49**	**33**	< 0.001**
**IVC(cm)**	**Max**	**1.71 ± 0.49**	**38**	**1.29 ± 0.21**	**33**	< 0.001**
	**Min**	**1.002 ± 0.54**	**36**	**0.63 ± 0.13**	**33**	< 0.001**

Parameters	Right –sided valvular lesion involvement-PRESENT (Mean ± SD) (N = 38)	Right –sided valvular lesion involvement-ABSENT (Mean ± SD) (N = 34)	p value
LV GLS (%)	− 16.73 ± 4.49	− 18.35 ± 3.53	0.092
RV free wall strain (basal,mid,apical) (%)	− 18.37 ± 6.67	− 20.75 ± 5.17	0.094
LV Peak systolic strain (%)	− 21.78 ± 27.89	− 17.27 ± 4.09	0.332
RV Peak systolic strain (%)	− 17.01 ± 6.79	− 19.52 ± 5.98	0.100
LV SSR(s^−1^)	− 4.84 ± 15.04	− 1.26 ± 0.81	0.152
RV SSR(s^−1^)	− 1.26 ± 0.45	− 1.42 ± 0.34	0.076
LV EDSR(s^−1^)	1.63 ± 0.99	1.65 ± 2.11	0.972
RV EDSR(s^−1^)	1.51 ± 0.81	1.33 ± 0.53	0.261
LV LDSR(s^−1^)	0.89 ± 0.34	0.85 ± 0.39	0.606
RV LDSR(s^−1^)	1.28 ± 0.79	1.43 ± 0.61	0.392

*LV EDD* Left ventricular end diastolic dimensions, *LV ESD* Left ventricular end systolic dimensions, *LVEDV* Left ventricular end diastolic volume, *LVESV* Left ventricular end systolic volume, *LV EF* Left ventricular ejection fraction, *LV FS* Left ventricular fractional shortening, *LV ALEF* Left ventricular area-length ejection fraction, *LA* Left atrium, *TAPSE* Tricuspid annular pre-systolic excursion, *RVSP* Right ventricular systolic function, *IVC* Inferior vena cava, *GLS* Global longitudinal strain, *SSR* Systolic strain rate, *EDSR* Early diastolic strain rate, *LDSR* Late diastolic strain rate, *LV* Left ventricle, *RV* Right ventricle, *statistically significant

Bold signifies that they are statistically significant

Also, when tissue deformation imaging parameters for LV and RV were assessed to see any association with right-sided valvular lesions (tricuspid or pulmonary valve disease), none of them showed a statistically significant result.

## Discussion

In the present study, 72 individuals (which included 34 males and 38 females) were enrolled after confirming that they had at least moderate forms of multivalvular disease involving any 2 or more cardiac valves. Out of these 72 subjects, 11 of them had already undergone percutaneous balloon mitral valvulotomy (BMV) for mitral stenosis but still had persisting mild to moderate degrees of mitral stenosis.The demographic and clinical features of the patients are given in Table 1.

Rheumatic heart disease (RHD) defined as a manifestation of rheumatic fever, an autoimmune disorders affecting multiple organ systems, is a disease associated with poor hygiene, overcrowding, poverty and low socioeconomic status [6–9]. In India alone, there has been drastic variations in prevalence of RHD in different demographic regions. In the present study majority of subjects enrolled where illiterate, unemployed and from rural dwelling.

In 1943, Don Carlos et al. [21] reported that “Diet is a predisposing factor for rheumatic fever” collecting data from many countries including India. Rinehart et al. [22], 1934 reported that there is a belief that deficiencies in vitamin D, calcium and phosphorus(less intake of milk, eggs and fish) can predispose to development of rheumatic manifestations during infancy. He believed that the autoantibodies generated in rheumatic fever are a part of macrophage system in the body that are all formed from the mesodermal layer that also gives rise to bones and connective tissue; hence, deficiency of vitamin D, calcium and phosphorus can all make one’s body defense system weak.

However, in the present study, majority of individuals with MVD were non vegetarians, 49(68.1%) rather than vegetarians, 23(31.9%) (Table 1). Hence, concluding that diet alone is not a factor for development of RHD, further research is required in this field.

### Rheumatic heart disease and multivalvular heart disease

In this study, a population of significant multivalvular disease was enrolled, predominantly with rheumatic etiology(83.8%) and least were degenerative valvular disease(13.5%) (Table 1); unlike other studies carried out by Cramariuc et al., 2010 [23] and Song Y et al., 2013 [24], which included predominantly degenerative valvular diseases. Out of the 72 patients, majority of 38(52.8%) patients presented with left-sided valvular lesions (aortic and/or mitral) along with concomitant right- sided valvular lesions (tricuspid/pulmonary); remaining21 (29.2%) patients had aortic regurgitation and mitral regurgitation, 21(29.2%) patients had aortic regurgitation and mitral stenosis, 3(4.2%) patients had aortic stenosis and mitral regurgitation, 2(2.8%) had aortic stenosis and mitral stenosis respectively (Fig. 2).

In the Indian subcontinent, as discussed earlier RHD is the commonest cause of MVD accounting for about 2–4 million cases, as reported by Kumar et al., 2013 [25].As MVD remains to be the main setting in RHD, evaluation and management are challenging. There are multiple combinations possible in MVD and hence this variability leads to availability of limited data for management strategies. Although as each case of MVD differs from the other, we need to consider the underlying etiology, pathophysiological presentations, assess clinical features and echocardiographic parameters to provide the best treatment strategy [1, 10–12].

### Left and right ventricular strain patterns in MVD

In the present study, we have used both conventional echocardiographic parameters along with tissue deformation imaging parameters to determine different LV and RV strain patterns in MVD.Echocardiography being the main diagnostic tool for MVD, there are few pitfalls in considering 2D echocardiographic parameters alone, also it becomes cumbersome when measurements of more than one cardiac valvular involvement has to be ruled out [1, 10].There are many studies that have been conducted to evaluate myocardial function in valvular heart disease using the advanced 2D strain echocardiography, but there is very limited data focusing its application in MVD.

A recent study conducted by Andrew W et al., 2021 [26] considered necessity of strain echocardiography in subjects with mixed aortic valve disease (AS with AR), it was concluded that LV global strain had prognostic associations with clinical outcomes when left ventricular ejection fraction was normal; however, the author also focused on the fact that LV-GLS needs to consider which valvular lesion is the cause for LV remodeling when interpreting GLS,due to more complex relationship among mixed aortic valve disease as opposed to isolated AS or AR.

A study conducted by In-Jeong Cho et al., 2020 [27] was based on “Determinants of clinical outcomes in patients with mixed mitral valve disease”. They only assessed routine echocardiographic parameters to determine the clinical outcomes, strain echocardiographic parameters of LV were not assessed. They concluded that transmitral pressure gradient should be considered as an important echocardiographic parameter to predict the clinical outcomes.

Alex Felix et al., 2018 [15] conducted a study to evaluate right ventricular function in subjects with left-sided valve lesions, but patients with severe TR were excluded from the study as it could interfere during RV strain measurements. They concluded that RV function was depressed in these patients, and hence RV function assessment should also be considered in patients with left-sided valvular lesions. None, of the above mentioned studies used strain echocardiography for different presentations of MVD.

In the present study, we included patients with TR as well, because most of our patients with MVD had significant right-sided valvular disease with either aortic and/or mitral disease (38 patients out of 72) (Fig. 2). Also, it was observed that LV ejection fraction (63.83 ± 4.84.

%), other LV echocardiographic parameters and even strain parameters (LV-GLS: 17.49 ± 41.17%) were normal (Table 3). This may be due to various presentations of LV loading conditions, wall stress and LV remodeling condition based on the underlying presentation of MVD. However, unlike LV echocardiographic parameters, the RV strain parameters like RV free wall strain(basal,mid,apical) (− 19.49 ± 6.08%) showed reduced values (Table 3). This may be due to higher levels of pulmonary pressures and RV pressure overload secondary to left-sided valvular disease [13, 14].

### Involvement of right -sided valvular lesions with aortic or mitral valvular lesions

In the present study, out of 72 patients with MVD, 38 of them had right—sided valvular disease (moderate to severe tricuspid valve disease) with mitral and/or aortic valve disease. When RV echocardiographic parameters were compared 2D derived left atrial dimensions (p = 0.034) was significant; TAPSE (p < 0.001), RVSP (p < 0.001) and IVC dimensions (p < 0.001) showed highly statistically significant result (Table 2), whereas none of the tissue deformation imaging derived ventricular (LV and RV) strain parameters were statistically significant.

Mittal et al., 2001 [17] performed a study on “Echocardiographic assessment of RV function in patients with pure mitral stenosis”, they did not find any correlation between right ventricular systolic function and MS.

Another study by Galli et al., 2013 [16] studied 200 patients with degenerative AS, these patients showed RV dysfunction by echocardiography.

Later, in literature many studies were done to assess RV function by echocardiography in left –sided valvular lesions, whereas none included MVD.They concluded that the impairment of RV function in left-sided valvular disease is either due to increased pulmonary capillary pressures, RV overload, ventricular interdependence (due to septal flattening because of RV enlargement) and due to some inflammatory changes due to rheumatic disease in RHD cases [15–20].

## Limitation of the study

Unequal gender distribution and multivalvular disease type is the major limitation in our study due to constricted time limit. Smaller sample size due to ongoing COVID pandemic, this may have limited our ability to detect significant associations with other factors in MVD subjects.

## Clinical implications

Despite of having any presentation of MVD, the prognosis and course of treatment should be based on approach to individual valvular lesions, as the use of strain echocardiographic parameters alone is not sufficient.

## Conclusion

We have provided new insights about alterations in ventricular strain patterns for different combinations of MVD. Subclinical myocardial dysfunction assessment using strain echocardiography is again dependent on various loading conditions on the ventricles and severity of the lesions. Also, it is extremely important that RV strain analysis should even be employed for MVDs affecting the left-sided heart valves alone.

## Data Availability

Master sheet with individual patient data will be submitted if requested.

## Abbreviations

MVD: Multivalvular disease
LV: Left ventricle
RV: Right ventricle
GLS: Global longitudinal strain
TAPSE: Tricuspid annular plane systolic excursion
RVSP: Right ventricular systolic pressure
IVC: Inferior vena cava
RHD: Rheumatic heart disease
AS: Aortic stenosis
AR: Aortic regurgitation
MS: Mitral stenosis
MR: Mitral regurgitation
RCM: Restrictive cardiomyopathy
IVS: Interventricular septum
EZR: Easy R statistical software
